# CRISPR-Cas9: A Revolutionary Tool for Cancer Modelling

**DOI:** 10.3390/ijms160922151

**Published:** 2015-09-14

**Authors:** Raul Torres-Ruiz, Sandra Rodriguez-Perales

**Affiliations:** 1 Viral Vector Technical Unit, Fundacion Centro Nacional de Investigaciones Cardiovasculares (CNIC), Melchor Fernandez Almagro, 3, 28029 Madrid, Spain; 2 Molecular Cytogenetics Group, Human Cancer Genetics Program, Centro Nacional de Investigaciones Oncológicas (CNIO), Melchor Fernandez Almagro, 3, 28029 Madrid, Spain

**Keywords:** CRISPR-Cas9 system, cancer modelling, gene mutations, rearrangements, genome engineering

## Abstract

The cancer-modelling field is now experiencing a conversion with the recent emergence of the RNA-programmable CRISPR-Cas9 system, a flexible methodology to produce essentially any desired modification in the genome. Cancer is a multistep process that involves many genetic mutations and other genome rearrangements. Despite their importance, it is difficult to recapitulate the degree of genetic complexity found in patient tumors. The CRISPR-Cas9 system for genome editing has been proven as a robust technology that makes it possible to generate cellular and animal models that recapitulate those cooperative alterations rapidly and at low cost. In this review, we will discuss the innovative applications of the CRISPR-Cas9 system to generate new models, providing a new way to interrogate the development and progression of cancers.

## 1. Introduction: The Evolution of Cancer Modelling

Cancer is a multiple-hit disease caused by mutations in genes involved in the control of cell function, especially those that control cell growth and division. It enables cells to grow and divide without the need for growth signals and to evade normal checks and balances [1]. Improvements in whole-genome sequencing have generated abundant data and detailed sequencing of human cancer cells in particular has revealed the complexity of the cancer genome, which undergoes numerous point mutations and large genome rearrangements [2,3]. These data must now be converted into functionally and clinically relevant knowledge. In this scenario, the generation of cellular and animal models to validate new candidate cancer genes and to gain insights into the molecular mechanisms underlying tumorigenesis is indispensable. Cancer models have evolved over the years through increasingly complex phases, including: (i) cell lines established from human tumors; (ii) xenografts derived from explants or cell lines; and (iii) transgenic or endogenous genetically-engineered cell or animal models [4].

Genes can be manipulated using several techniques to create genetically-engineered cell or animal models. Traditionally, engineered cancer models were obtained by exogenous expression of transgenes or manipulation of genes by homologous recombination [5]. During the last ten years, more accurately programmable nucleases have been developed for manipulating genomes, thus enabling a more precise and sophisticated approach to genetic modelling [6]. These tools include zinc-finger nucleases (ZFNs) and transcription-activator-like effector nucleases (TALENs) [7,8]. However, the recent advent of the clustered regularly-interspaced short palindromic repeats (CRISPR)-Cas9 system has revolutionized the field of cancer modelling. Unlike the genome-editing tools ZFNs and TALENs, which are based on sequence recognition via protein–DNA interactions, the CRISPR-Cas9 system can target specific genomic loci with a single-stranded guide RNA. Thus, reprogramming of Cas9 is simplified, especially taking into account that RNA is much easier to synthesize and to introduce into the cell than protein domains. This approach facilitates targeted genome modifications. As mentioned above, CRISPR-Cas9 relies on the expression of a small guide RNA (sgRNA). When coupled with a Cas9 nuclease, the sgRNA can potentially target any locus in the genome by generating double-strand breaks (DSBs) that stimulate cellular DNA repair mechanisms, including error-prone non-homologous end joining (NHEJ) and homology-directed repair (HDR) [9]. This technique has been adapted to engineer the genome in a wide range of experimental models (e.g., human, mouse, rat, zebrafish, fruit fly and rhesus monkey) [10]. The CRISPR-Cas9 system can tolerate mismatches in the DNA target with respect to the sgRNA sequence, thus potentially promoting damage at off-target sites [11]. However, the combination of pairs of Cas9 nickase with pairs of sgRNAs prevents this problem from arising [12,13].

Cell and animal models are key to increase our understanding of tumor biology [14] and represent powerful preclinical platforms for testing of new compounds [15,16]. In this review, we describe the novel strategies and mechanisms that have been used to generate CRISPR-Cas9-engineered cancer models and highlight key challenges in the application of these sophisticated approaches.

**Table 1:** Overview of the use of the CRISPR-Cas9 system in the context of cancer modelling. HSPC, hematopoietic stem progenitor cells.

Approach	Alteration	Target Cell	Disease	Gene	Delivery	Reference
*in vitro*	Loss-of-function	mHSPC (mouse)	Acute myeloid leukemia (AML)	*Mll3*	Plasmid transfection	Chen *et al.* (2014) [17]
*in vitro*	Loss-of-function	JygMC cell line (mouse)	Triple-negative breast cancer (TNBC)	*Cripto-1*	Plasmid transfection	Castro *et al.* (2014) [18]
*in vitro*	Loss-of-function	mHSPC (mouse)	Acute myeloid leukemia (AML)	*Tet2*, *Runx1*, *Dnmt3a*, *Ezh2*, *Nf1*, *Smc3*, *p53*, *Asxl1*	LV	Heckl *et al.* (2014) [19]
*in vitro*	Loss-of-function	A375 melanoma cell line (human)	Melanoma	GeCKO library	LV	Shalem *et al.* (2014) [20]
*in vitro*	Loss-of-function and directed mutation	DLD1 and HCT116 cell lines (human)	Colon cancer	*PKC*	Plasmid transfection	Antal *et al.* (2015) [21]
*in vitro*	Loss-of-function and directed mutation	Organoids intestinal epithelium (human)	Colorectal tumor	*APC*, *SMAD4*, *TP53*, *KRAS*, *PIK3CA*	Electroporation	Matano *et al.* (2015) [22]
*in vitro*	Loss-of-function and directed mutation	Organoids intestinal epithelium (human)	Colorectal tumor	*APC*, *SMAD4*, *TP53*, *KRAS*	Electroporation	Drost *et al.* (2015) [23]
*in vitro*	Gain-of-function	A375 melanoma cell line (human)	Melanoma	SAM library	LV	Konermann *et al.* (2014) [24]
*in vitro*	Chromosomal rearrangement	HEK293, hMSC, hHSPC (human)	Ewing sarcoma, acute myeloid leukemia	*EWSR1-FLI1*, *RUNX1-ETO*	Plasmid transfection	Torres *et al.* (2014) [25]
*in vitro*	Chromosomal rearrangement	HEK293 (human)	Lung adenocarcinoma	*CD74-ROS1*, *EML4-ALK*, *KIF5B-RET*	Plasmid transfection	Choi and Meyerson (2014) [26]
*in vitro*	Chromosomal rearrangement	HCT116 cell line (human)	na	*NPM1-ALK*	Plasmid transfection	Ghezraoui *et al.* (2014) [27]
*in vitro*	Chromosomal rearrangement	Myoblasts (mouse)	Alveolar rhabdomyosarcoma (A-RMS)	*Pax3-Fkhr*	LV	Lagutina *et al.* (2015) [28]
*in vitro*	Deletion and knock in	hESC (human)	na	*TERT*	Electroporation	Chiba *et al.* (2015) [29]
*in vivo*	Loss-of-function	Lung cells (mouse)	Lung adenocarcinoma	*Nkx2.1*, *Pten*, *Apc*	LV	Sanchez-Rivera *et al.* (2*0*14) [30]
*in vivo*	Loss-of-function	Somatic pancreatic cells (mouse)	Pancreatic ductal adenocarcinoma (PDAC)	*Lkb1*	LV and AdV	Chiou *et al*. (2015) [31]
*in vivo*	Loss-of-function	Cell line (mouse)	Non-small-cell lung cancer (NSCLC)	GeCKO library	LV	Chen *et al.* (2015) [32]
*in vivo*	Loss-of-function and directed mutation	mESCs and zygotes (mouse)	na	*Tet1*, *Tet2*, *Tet3*, *Sry*, *Uty*	Injection	Wang *et al.* (2013) [33]
*in vivo*	Loss-of-function and directed mutation	Neurons, immune and endothelial cells (mouse)	Lung adenocarcinoma	*Kras*, *p53*, *Lkb1*	AAV, LV and particle-mediated delivery	Platt *et al.* (2014) [34]
*in vivo*	Loss-of-function and directed mutation	Liver cells (mouse)	Liver cancer	*Pten*, *p53*	Injection	Xue *et al.* (2014) [35]
*in vivo*	Chromosomal rearrangement	Embryo (zebrafish)	na	*mir-126a*	Injection	Xiao *et al.* (2013) [36]
*in vivo*	Chromosomal rearrangement	Lung cells (mouse)	Non-small-cell lung cancer (NSCLC)	*Eml-Alk*	LV	Blasco *et al.* (2014) [37]
*in vivo*	Chromosomal rearrangement	Lung cells (mouse)	Non-small-cell lung cancer (NSCLC)	*Eml-Alk*	AdV	Maddalo *et al.* (2014) [38]

mHSPC: mouse haematopoietic stem/progenitor cell; LV: lentivirus; hMSC: human primary mesenchymal stem cells; hHSPC: human haematopoietic stem/progenitor cell; hESC: human embryonic stem cells; na: not applicable; AAV: adeno-associated virus; AdV: adenovirus.

## 2. Generation of CRISPR Cancer Models

In order to model the pathogenesis of cancer, it is necessary to efficiently reproduce the complex genetic scenario associated with tumorigenesis in specific cell types or organisms [39,40]. Traditional experimental approaches used to model the altered genome characteristic of oncogenic cells are limited by their complex design and run time. CRISPR is an efficient and accurate tool that has facilitated rapid genetic modification and made genetic engineering streamlined and widely applicable, both for generating precise human cellular models and for increasing the ease and effectiveness of cancer models in other species [41,42] (Table 1). The advances made can improve our understanding of cancer biology and facilitate drug screening and the search for new therapies.

### 2.1. Modelling Oncogenic Alterations in Vitro

Human tumor-associated processes have a common major driving force, namely genome alterations [43]. Alterations that include large chromosomal rearrangements (*i.e*., translocations, duplications, deletions or inversions) and point mutations lead to inactivation of tumor suppressor genes (TSGs), activation of oncogenes and alteration of genes involved in repair processes. CRISPR-Cas9 has become the tool of choice for mimicking these scenarios, not only because of its ease of application, but also because of its efficiency and accuracy.

#### 2.1.1. *In Vitro* Modelling of Chromosomal Rearrangements

The initial event in the generation of large chromosomal rearrangements is the co-occurrence of two DNA double-strand breaks (DSB) in the genome of a cell. Sometimes, the broken ends are recognized and repaired by cellular repair machinery to form a rearranged configuration, by which chromosomal translocations, deletions, inversions and amplifications are generated. These aberrantly-repaired DNA products can act as driver or passenger events in oncogenesis [44]. Different adaptations of the CRISPR system have enabled accurate reproduction of large chromosomal rearrangements *in vitro* by means of two sgRNAs targeting the loci involved in a specific genomic event. The CRISPR-Cas9 system has considerably improved the efficiency of conventional approaches and ZFNs or TALENs [45].

In 2014, our group [29] reported that the adaptation of the CRISPR-Cas9 system to generate targeted cancer chromosomal translocations in human cells has paved the way for the generation of cellular models that recapitulate the primary oncogenic events driving tumorigenesis. The approach was based on a pair of plasmids expressing Cas9 and two sgRNAs that targeted the breakpoints of a cancer translocation. We generated and characterized the Ewing sarcoma hallmark t(11;22)/*EWSR1-FLI1* chromosomal translocation in the HEK293 cell line and in human primary mesenchymal stem cells (hMSC), with efficiencies of 1.76% and 0.15%, respectively. The aberrant EWSR1-FLI1 transcription factor, which is a consequence of the *de novo* chromosomal translocation, altered the expression of known downstream target genes, thus mimicking the effect observed in human tumors. We also demonstrated the universalization of this approach by reproducing the acute myeloid leukemia (AML) t(8;21)/*RUNX1-ETO* chromosomal translocation in HEK293 and CD34+ human hematopoietic progenitor cells. Again, efficiency was remarkably higher in easily-transfectable established cell lines than in primary cells (4.07% and 0.5%, respectively). Almost in parallel, Choi and Meyerson [30] reported the application of the CRISPR-Cas9 system to generate a chromosomal translocation and two inversions involved in the development of lung cancer. The authors used a similar approach to mimic the lung adenocarcinoma chromosomal translocation t(5;6)/*CD74-ROS1* in HEK293 cells and in non-transformed immortalized lung epithelial cells (AALE). They also generated two types of inversion events, a paracentric *EML4-ALK* inversion with the breakpoints localized in the short arm of human chromosome 2 and a pericentric *KIF5B-RET* inversion across the two arms of chromosome 10. Both inversions were reproduced separately in the HEK293 cell line, yielding an efficiency of 8.9% for the paracentric inversion and 1.62% for the pericentric inversion. These and other results [11,29,30] seem to indicate that the adaptation of the CRISPR system to reproduce chromosomal rearrangements is clearly dependent on the localization of the breakpoints in that efficiency decreases as the distance between the breakpoints increases. This approach must be improved upon in order to facilitate the generation of the desired targeted product, especially in cases where the rearrangement itself does not confer any growth advantage.

Ghezraoui *et al*. used the ZFN, TALEN and CRISPR systems to elucidate the mechanisms of genome rearrangements involved in the accumulation of alterations in cancer cells [31]. The authors examined the joining mechanism involved in the generation of human chromosomal translocations, particularly the role of NHEJ pathways, used either the wild-type CRISPR system or paired Cas9 nickases to reproduce the t(2;5)/*NPM-ALK* translocation commonly found in anaplastic large cell lymphoma in the modified HCT116 cell line and obtained a targeted translocation efficiency of 0.13%. The study concluded that wild-type Cas9 leads to precise joining of the DSBs, whereas paired nickase-Cas9 produces insertion/deletions (indels) in the junction regions of the translocation derivative chromosomes. This controversial issue could be clarified in the near future using CRISPR combined with proper controls and methodologies.

The CRISPR-Cas9 system can be adapted to generate specific mutations or deletions in non-coding or promoter sequences. Chiba *et al*. [33] used this ability to understand the role of common *TERT* promoter alterations in many neoplastic processes. Using two flanking sgRNAs, the authors were able to induce cancer-associated *TERT* promoter deletions in human embryonic stem cells (hESCs). Initially, these changes did not promote any effect in hESCs (no significant difference in telomere length was observed compared to control cells). However, upon differentiation into somatic cells, which normally silence telomerase, the mutation leads to unsilencing of the telomerase gene, showing levels of expression comparable to those of cancer cells and aberrantly long telomeres.

#### 2.1.2. Modelling Targeted Mutations

The ability to induce targeted genome modifications in somatic cells empowered the study of gene function in several areas of biology, particularly cancer biology.

In mid-2014, the CRISPR-Cas9 system was used in combination with a short hairpin RNA (shRNA) approach to study the role of 7q deletions in promoting leukemogenesis [21]. The authors used CRISPR to disrupt the *Mll3* gene in mouse hematopoietic stem progenitor cells (HSPC) in an *Nf1*- and *p53*-deficient genetic context. The analysis of the leukemic cells produced by engineering of those genes revealed that they were heterozygous for *Mll3*, suggesting that leukemogenesis is based on partial, but not complete inactivation of *Mll3* and supporting the idea that *Mll3* is a haploinsufficient TSG that may act as a second hit in many AML processes. In September of 2014 [23], the CRISPR-Cas9 system was first shown to induce multiple cooperating mutations, thus making it possible not only to induce a specific driving genetic mutation, but also to reproduce other secondary associated hits. The authors used a combined two-vector lentiviral approach to express CRISPR components in mouse HSPCs harboring Flt3-ITD to modify up to five genes *ex vivo* in a single cell clone. Cooperating loss-of-function mutations in genes recurrently inactivated in myeloid malignancies were introduced into HSPCs to create AML models. The genes included encoding epigenetic modifiers, transcription factors and mediators of cytokine signaling (*Tet2*, *Runx1*, *Dnmt3*, *Ezh2*, *Nf1*, *Smc3*, *p53* and *Asxl1*), thus mimicking the genetic combinations observed in patients with AML and leading to myeloid clonal expansion and transformation to acute leukemia. In January 2015, the CRISPR-Cas9 system enabled Newton, Brognard and coworkers [25] to establish that mutations in protein kinase C (*PKC*) genes generally acted as tumor suppressors, thus modifying previous knowledge and creating a shift towards therapies aimed at restoring the altered gene rather than acting against PKC. The authors used CRISPR-Cas9 in combination with single-stranded oligodeoxynucleotides (ssODNs) to correct a *PKCβ* gene mutation in a colon cancer cell line (DLD1) from a patient. The correction of the mutation restored protein function and reduced tumor size in a xenograft model, thus demonstrating that *PKCβ* confers a growth advantage in this specific tumor environment and has a tumor suppressive function.

Multiplexing makes it possible to study multiple parameter variations simultaneously and is the ideal method for modelling the many changes that take place in cancer. Furthermore, it can be applied in a specific cell type, an issue not addressed until recently. During the last two years, several authors have reported the use of the CRISPR-Cas9 system to engineer multiple targets simultaneously. Matano *et al.* [26] took advantage of this ability to model colorectal cancer on human intestinal organoids. The authors modelled five mutations that promote growth independently of niche signals, including TSG (*SMAD4*, *TP53* and *APC*) and oncogenes (*KRAS* and *PIK3CA*). First, to create loss-of-function mutations in the human intestinal cells, a vector expressing Cas9 and one sgRNA was electroporated in human intestinal stem cells, and different combinations of mutant cell lines were obtained using an elegant selection procedure based on the growth advantages of these edited cells in medium lacking or containing specific factors [46]. Second, the CRISPR system was used in combination with ssODNs to knock in the specific mutations *KRAS^G12V^* and *PIK3CA^E545K^*, and the cells were selected again by adding or removing growth factors from the culture medium. This system enabled the authors not only to edit five genes in these organoids, but also to obtain single cell clones with a different mutational background that recapitulated the adenocarcinoma transition. Two months later, Drost *et al.* [27] published similar results in a study of the same genes, with the exception of *PIK3CA*, in the same cell type (human intestinal organoids).

#### 2.1.3. CRISPR-Cas9 High-Throughput Genetic Screens

One of the most promising and exciting applications of the CRISPR-Cas9 system is the option of studying gene functions on a genome-wide scale, thus making it a valuable resource for high-throughput screening. This advantage has been specifically exploited for the systematic study of genes involved in cancer by the generation of sgRNA libraries targeting thousands of genes.

In 2014, Zhang’s group reported the results of two *in vitro* studies [24,28] in which a loss-of-function library and a genome-wide gain-of-function sgRNA library targeting human genes were used to identify involvement in resistance to vemurafenib, a targeted therapy for melanoma. In the first study, the authors used the CRISPR-Cas9 knockout (GeCKO) library, which targets more than 18,000 human genes, to test for genes whose loss is involved in resistance to vemurafenib, a BRAF inhibitor, and found previously validated genes, as well as new candidates. The second study described the use of a sgRNA library targeting all human RefSeq coding isoforms to screen for genes that, upon activation, confer resistance to vemurafenib. Using a different approach (activation *versus* silencing), the authors found previously validated and novel candidate genes that confer resistance to the drug.

More recently, Zhang and Sharp [36] used a genome-wide loss-of-function sgRNA library to systematically screen genes involved in metastasis. The authors mutagenized a non-metastatic non-small-cell lung cancer cell line derived from a mouse using the GeCKO library described above. The mutant cell pools were transplanted into mice and generated tumors. After deep sequencing, a set of genes consistently represented in all of the tumors produced was found to be involved in tumor growth and metastasis.

### 2.2. Modelling Oncogenic Alterations in Vivo: Editing the Genome of Embryonic Stem Cells and Embryos

Highly efficient editing of the genome in a range of experimental models (including mice) can be achieved by simple delivery of editing reagents to embryonic stem cells. However, an interesting advantage of the CRISPR-Cas9 system over conventional gene targeting technology [47] is that it can directly modify the zygote genome, thus saving costs and time in the creation of genetically-modified organisms and obviating the need for embryonic stem cells as an essential intermediate. Editing reagents can be delivered into the cell in five forms: sgRNAs, as either plasmid or ssRNA; and Cas9 nuclease, as plasmid, mRNA or protein. Various strategies have been described for the delivery of editing reagents, including pronuclear microinjection of the sgRNA/Cas9 plasmid in a manner essentially identical to that used for generating transgenic mice or injection of Cas9 mRNA or protein directly into the cytoplasm or pronucleus, respectively. The strategies have advantages and disadvantages that have to be taken into account when choosing a particular delivery method, such as integration of the plasmid in a subset of embryos and differences in survival and targeting efficiency [48,49]. Another advantage of the CRISPR-Cas9 system is that it enables simultaneous mutation of multiple loci, thus improving the traditional generation of mice carrying mutations in multiple genes by sequential recombination of mouse embryonic stem cells (mESCs) or by intercrossing of mice with a single mutation.

In 2013, Wang *et al.* [37] established three CRISPR-Cas9-based approaches for the generation of mice carrying multiple genetic alterations. The first involved co-transfection of mESCs with pooled constructs expressing Cas9 and sgRNAs. This strategy enabled the induction of multiplexed gene depletion. In the first round of experiments, 20.8% of the clones tested showed mutations in the six alleles of the *Tet1*, *2* and *3* genes. The authors then tried to knock out five genes (*Tet1*, *2*, *3*, *Sry* and *Uty*) and reported that 10% of the clones had a depletion in eight alleles of the five genes. In the second approach, the authors directly modified mouse embryos by injecting Cas9 mRNA and sgRNAs into the fertilized egg. Co-injection of Cas9 and a single sgRNA for the *Tet1*, *2* or *3* genes into zygotes led 89% of mice to carry indel mutations in a single gene. When a combination of two sgRNAs targeting *Tet1* and *Tet2* were used, 70% of mice born showed indels in both genes. In the third approach, the authors generated mutant alleles with predetermined alterations in zygotes by co-injection of Cas9 mRNA and sgRNA and single-stranded DNA (ssODNs) with a desired mutation. This approach made it possible to generate, in a single step, one or two pre-designed *Tet1* and *Tet2* point mutations in around 60% and 7% of mice, respectively. The study by Wang *et al*. [37] paved the way for systematic genome-engineered mice and was particularly useful in that it enabled the authors to produce mice carrying multiple alterations in loci thought to play a role in the genesis of multigenic diseases, such as cancer.

In cases where indels do not disrupt function, such as non-coding genes or regulatory sequences, large genomic deletions or inversions are needed. Xiao *et al*. [40] described the use of TALEN and CRISPR to obtain predictable genomic deletions or inversions with sizes ranging from several hundred bases to nearly 1 Mb. The injection of Cas9 mRNA and two sgRNAs targeting distal DNA sites of the same chromosome in zebrafish embryos yielded animals with a predictable genomic deletion. For the first time, the CRISPR strategy made it possible to delete genomic regions containing the dre-mir-126a or miRNA cluster Chr.9 in the zebrafish genome (1%–3% of cases).

### 2.3. Modelling Oncogenic Alterations in Vivo: Adult Animals

The generation of genetically-engineered animal models that recapitulate genomic mutations or chromosomal rearrangements using classic approaches is expensive and time consuming, since it requires embryonic stem cell engineering and mutant animal crossbreeding [50]. Recent studies have demonstrated that the CRISPR-Cas9 system can generate sophisticated cancer models targeting the somatic cells of adult animals [42]. This method allowed for faster *in vivo* testing of single genes or combinations of genes in different target tissues when used in combination with efficient delivery techniques, such as adenoviruses or adeno-associated virus [51]. The advantages of this approach include the possibility of targeting several loci to model the effects of cooperative genetic events or of using different cancer-relevant mouse genetic backgrounds in the initiation and progression of cancer.

The first study in which the CRISPR-Cas9 system was successfully used for precise editing of the genome of adult somatic cells was published in October 2014 [39]. Xue *et al.* [39] targeted a single gene or a simultaneous combination of two genes in adult mice hepatocytes by delivering a plasmid for transient expression of Cas9 and sgRNAs through hydrodynamic tail vein injection. The authors used this strategy to create loss-of-function mutations in *Pten* (4%), *p53* (6.4%) or both genes. Three months later, all mice inoculated with sgRNAs simultaneously targeting *Pten* and *p53* developed liver tumors. These results illustrate that CRISPR is a potent strategy for modelling carcinogenic mutations similar to that of traditional genome-edited Cre/*loxP* controls. The study also reports the induction of *Pten* mutations using the safer off-target CRISPR nickase approach, albeit with lower efficiency (2.7%)*.* Finally, the authors demonstrated that co-delivery of a CRISPR expression plasmid targeting the *Ctnnb1* gene and single-stranded DNA could be used to directly induce gain-of-function mutations via homologous recombination *in vivo* with 0.5% efficiency.

Another approach for multigene interactions was described by Platt *et al.* [38], who generated a Cre-dependent Cas9 mouse by inserting a floxed-stopped Cas9 expression cassette into the *Rosa26* locus*.* The first step was to demonstrate that the constitutive or tissue-specific expression of Cas9 produced no adverse effects in the mouse. The authors then induced consecutive multiple genetic lesions in the same Cre-dependent Cas9 mouse by intranasal or intratracheal delivery of a single serotype9 adeno-associated virus (AAV) for the expression of the three sgRNAs, an HDR donor template with the *Kras^G12D^* mutation and Cre recombinase. Multiplex sgRNA delivery was used to simultaneously target the oncogene *Kras* and the tumor-suppressor genes *p53* and *Lkb1*. Two months post-infection, 100% of the treated animals had generated a spectrum of tumors in the lungs. This model enabled closer recapitulation of the accumulation of mutations in lung cancer by the generation of the *Kras^G12D^* mutation and knock-out of *p53* and *Lkb1* genes in the Cre-dependent Cas9 mouse.

Blasco *et al.* [41] showed the CRISPR-Cas9 system to be a feasible approach not only for the generation of gene mutations, but also for *in vivo* engineering of oncogenic chromosomal rearrangements in mice and, potentially, other species*.* The authors reported the successful induction of the *Eml4-Alk* gene rearrangement found recurrently in non-small-cell lung cancers. Lentiviral particles were used for intratracheal or intrapulmonary inoculation of the CRISPR components to adult mouse lung tissue. The *Eml4* and *Alk* genes, both of which are localized in mouse chromosome 17, were targeted by two sgRNAs inducing two DSBs in the same chromosome, which, in some cases (1.5 rearrangements/10^6^ cells), were repaired by generating a 10-Mb inversion. Almost in parallel, Maddalo *et al.* [42] reported the induction of the same *Eml4-Alk* inversion in lung somatic cells of adult mice. However, the authors used adenoviral-mediated delivery of the CRISPR system by intratracheal instillation to deliver Cas9 and sgRNAs targeting the *Alk* and *Eml4* loci in the lungs. In both studies, two to three months after inoculation, all mice developed lung tumors expressing the Eml4-Alk fusion oncogene and displaying histopathological and molecular features typical of ALK-positive human non-small-cell lung cancer.

Sanchez-Rivera *et al.* [34] demonstrated the power of combining the CRISPR system with Cre-*loxP* conditional cancer mouse models [34]. The authors generated a lentivirus-based CRISPR approach to express Cre recombinase and CRISPR components (sgRNA and Cas9) for rapid functional investigation of genes in the context of conditional adult mouse cancer models. As test models, they chose genetically-engineered mouse models of lung adenocarcinoma with *Kras*^G12D/+^- or Cre-dependent *p53*^flox-flox^ and analyzed the effect of the induction of loss-of-function mutations in *Nkx2.1*, *Pten* and *Apc* TSGs upon intratracheal administration of the lentiviral vectors. The results demonstrated that the system is highly efficient *in vivo*, leading to significant histological and pathway-specific differences upon deletion of each of those three TSGs in lung tumors. CRISPR is a rapid somatic genome engineering platform for functional characterization of putative cancer genes in the context of established mouse models.

Chiou *et al.* [35] recently reported the generation of a Cre-regulated Cas9 mouse by integration of an LSL-Cas9 cassette into the mouse *H11* locus. In those mice, Cas9 will only be expressed after recombination of the Stop cassette induced by the expression of Cre recombinase. Direct inoculation by retrograde pancreatic ductal injection of an adenoviral or lentiviral vector was used to express Cre, and a sgRNA was used to target the *Lkb1* gene in the pancreas. CRISPR-mediated targeting of Lkb1 led to rapid tumor growth that phenocopied Cre-mediated genetic deletion of the *Lkb1* gene. This approach could potentially be used *in vivo* to inactivate genes of interest in pancreatic cancer without the need to generate new mouse alleles.

## 3. Current Challenges and Potential Solutions

Although CRISPR-Cas9 has been successfully applied to model cancer using many different approaches, the system is still subject to technical limitations. One of the main challenges, both *in vivo* and *in vitro*, concerns delivery of the necessary components to the target cell. Many efforts have been made to increase the efficiency of delivery and to broaden its applicability. Since traditional plasmid delivery methods were first used [52,53], two additional approaches have evolved: mRNA [54,55] and Cas9 ribonucleoproteins (RNP) [56] (*in vitro* transcribed sgRNA complexed with recombinant Cas9 protein), which seem to show the most promising results in poorly transfectable cells [57]. However, this approach is limited by the fact that it cannot be used *in vivo*, because RNP cannot be delivered with adequate efficiency to target cells in a living organism. Use of viral vectors seems more practical, and several attempts have been made in this respect [58,59,60,61]. These methodologies reach a broad range of cells, especially primary adult stem cells, which are very difficult to modify using other methods. Consequently, the combination of a gene-editing tool and delivery method is more powerful, although it has to take into account the possible off-target effects derived from the use of both together. In many of the studies reviewed here, lentiviruses were the first choice. Lentiviruses are able to reach almost any cell type [62,63] and to generate a broad spectrum of approaches in combination with modifying enzymes (nucleases, nickases and recombinases [64,65,66]), meigaialthough they have mutagenic potential that is intensified when they are combined with designer nucleases. Adenoviruses have also been used [58] and lead to more specific genome editing. However, they are less efficient and difficult to apply *in vivo*. Their widespread use is hampered because they are more difficult to generate than conventional lentiviruses. Adeno-associated viruses are one of the most promising alternatives, especially owing to their retargeting ability [67], although they do have a major drawback, namely their limited packaging size (4.7 kb) [68].

The CRISPR-Cas9 system can induce off-target mutations at sites that are highly homologous to on-target sites [11,69,70,71,72]. Off-target DNA cleavage can cause unwanted genetic changes and/or chromosomal rearrangements with unpredictable consequences [11]. This caveat, which is a major concern in original *in vitro* studies, seems less relevant *in vivo* [73,74,75,76,77]. Further studies are required to fully elucidate the side effects of the CRISPR system.

**Figure 1:**
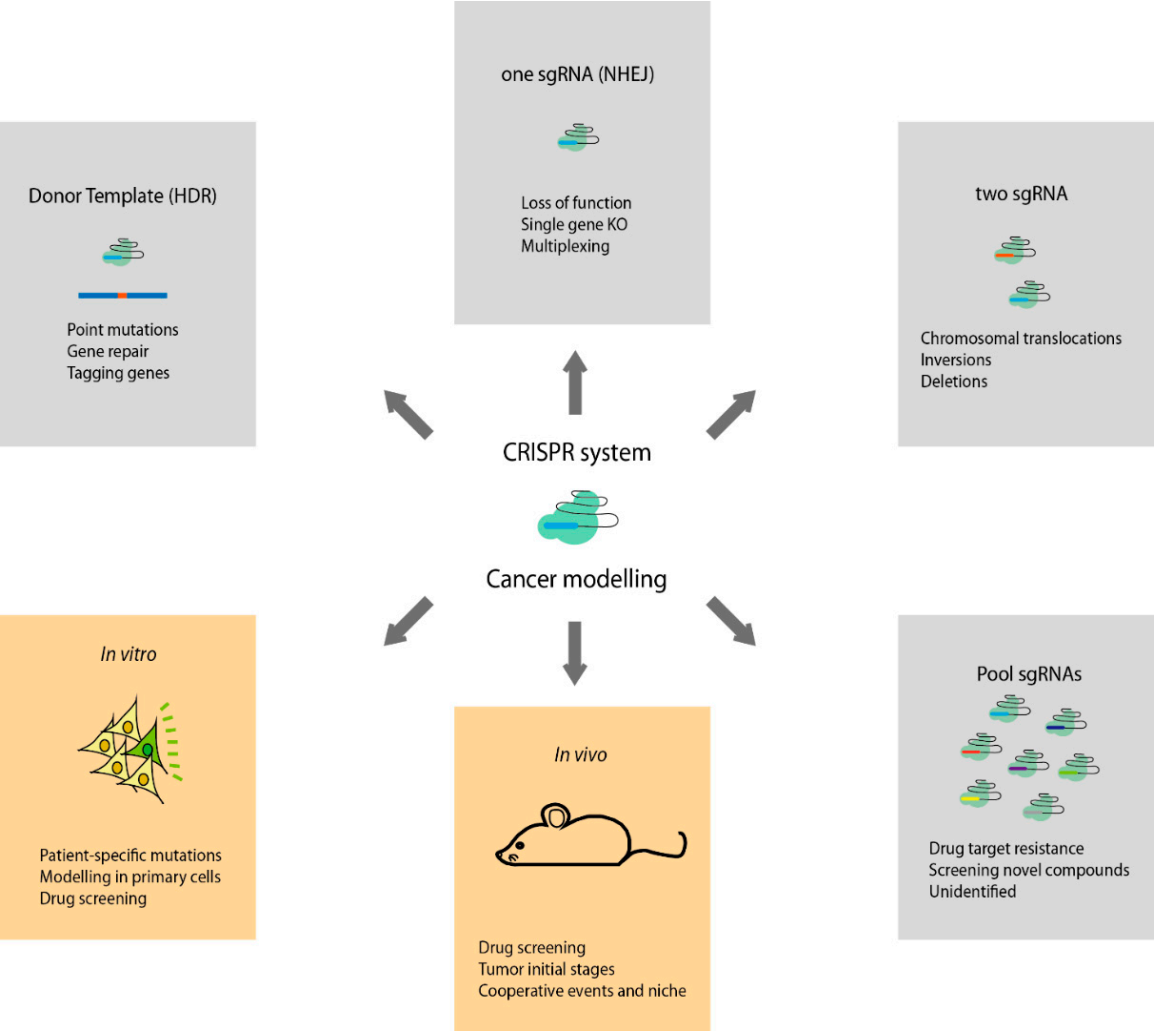
Types of genome-engineering CRISPR-Cas9 system applications to study cancer. HDR: homology-directed repair; sgRNA: small guide RNA; NHEJ: non-homologous end joining.

The studies reviewed in the present article clearly demonstrate the high efficiency of the CRISPR system in the modification, repair and deletion of genes using either *in vitro* or *in vivo* approaches (Figure 1). However, although CRISPR is a promising technique for cell therapies targeting cancer genes in humans, its potential has yet to be fully determined. Off-target effects give cause for concern, with the result that more in-depth studies must be performed and new strategies tried to ensure that only desired genes are edited and that the effects of the use of CRISPR in human somatic cells is known. In addition, ethical issues associated with engineering the human germline are sure to play a role in the future [78]. Nevertheless, there is no doubt that the potential of CRISPR in human medicine more than compensates for the concerns that arise.
